# Societal implications of expanded universal carrier screening: a scoping review

**DOI:** 10.1038/s41431-022-01178-8

**Published:** 2022-09-12

**Authors:** Lieke M. van den Heuvel, Nina van den Berg, A. Cecile J. W. Janssens, Erwin Birnie, Lidewij Henneman, Wybo J. Dondorp, Mirjam Plantinga, Irene M. van Langen

**Affiliations:** 1 Department of Genetics, University Medical Center Groningen, University of Groningen, Groningen, The Netherlands; 2 Department of Human Genetics and Amsterdam Reproduction and Development research institute, Amsterdam UMC, Vrije Universiteit Amsterdam, Amsterdam, The Netherlands; 3 Department of Epidemiology, Rollins School of Public Health, Emory University, Atlanta, GA USA; 4 Department of Health, Ethics & Society, Research Schools CAPHRI and GROW, Maastricht University, Maastricht, The Netherlands

**Keywords:** Genetic testing, Medical ethics

## Abstract

Carrier screening aims to identify couples at risk of conceiving children with a recessive condition. Until recently, carrier screening was primarily offered ancestry-based. Technological advances now facilitate expanded universal carrier screening (EUCS). This scoping review aimed to map EUCS’s potential societal implications based on both theoretical studies and empirical evidence. To this aim, we performed a CoCites search to find relevant articles, including articles describing carrier screening for at-risk populations, based on five selected query articles. Forty articles were included. Three main potential societal implications were identified: (1) unwanted medicalization, (2) stigmatization and discrimination of carriers and people affected with the conditions screened and (3) challenges in achieving equitable access. Within these themes, potential positive implications are reduction of ethnic stigmatization in ancestry-based offers and increased equity. Potential negative implications are reinforcement of disability-based stigmatization, less possibility for developing expertise in healthcare and societal pressure to partake in screening. Empirical evidence on all these implications is however scarce. In conclusion, both positive and negative potential societal implications of implementing EUCS, primarily theoretical, were identified, even in at-risk groups where evidence is mostly lacking. Empirical research in EUCS pilots is needed to identify which societal implications are likely to occur and therefore should be overcome when implementing EUCS.

## Introduction

Carrier screening provides prospective parents information about their carrier status for autosomal recessive and x-linked conditions. If couples prove to be carriers, they have a 1 in 4 risk in each pregnancy of having a child with an autosomal recessive condition. For X-linked recessive conditions, there is a 50% risk for male offspring to be affected if the woman is found to be a carrier. It is estimated that approximately 1 in 100/125 couples have a risk of having a child affected with a serious recessive condition [1]. Generally, information on carrier status is provided with the aim of informing reproductive decision-making. Prior to pregnancy (i.e., preconceptionally), the range of reproductive options after testing is wider than during pregnancy and may include preimplantation genetic testing or the use of donor gametes. This contrasts with prenatal carrier screening, where the only option is having or not having prenatal diagnosis. If then the child proves to be affected, a couple may decide to terminate the pregnancy or prepare for a child with the condition identified. Although offering carrier screening preconceptionally is widely regarded as ethically preferable, couples may often only be reached once a pregnancy is already established [2].

Until recently, carrier screening for one or a few conditions was mostly offered to at-risk populations based on ancestry or geographical origin [3]. In addition to the aim of informed reproductive decision-making, many of these programs were set up to prevent suffering of children and families in communities with a high disease burden and/or to prevent the society from having insufficient healthcare budgets to take appropriate care for those affected by the condition [4, 5]. Examples of these screening offers include carrier screening for Tay-Sachs disease among the Ashkenazi Jewish population, for example as part of the carrier-matching program Dor Yeshorim [6], and the semi-mandatory screening offer for beta-thalassemia on Cyprus [7].

Since approximately 2011, expanded carrier screening has come into practice as a reliable, fast, and relatively affordable screening method that allows for a population-based or universal offer to all couples with a child wish, regardless of their a priori risk [5]. The term “expanded carrier screening” (ECS) consists of different elements. As a form of screening, ECS entails a routine offer of medical testing to a target population of people without necessarily having a prior indication for such testing based on elevated risk for the conditions involved. The fact that the test looks for carrier status of recessive conditions makes it a form of carrier screening. The qualification ‘expanded’ refers to the scope of the screening test, which allows simultaneous testing for multiple recessive conditions [8, 9]. ECS can be available universally, allowing all couples with a desire to have children to partake. In several countries, such as United States, ECS is now routinely offered in a healthcare setting through use of commercial test offers [10]. Although many ECS and expanded universal carrier screening (EUCS) offers exist, including pilots carried out in several countries including the Netherlands [11] and Australia (“Mackenzie’s mission”) [12], EUCS has not been implemented as a population-based screening program anywhere in the world [13].

While EUCS potentially promotes reproductive autonomy for all couples planning to conceive, it also raises ethical, societal and psychological concerns, partly derived from concerns as well as experiences in at-risk populations [14]. To consider whether and how implementation of EUCS is desirable, and in what way(s) responsible implementation could be feasible, we believe it is important to map the (potential) implications of EUCS on the societal level [15, 16]. To our knowledge, this has not been done systematically before. This scoping review therefore aimed to answer the following research questions: (1) which (potential) societal implications of EUCS are described in the literature?, and (2) what is the (empirical) evidence available for these societal implications?

## Materials And methods

### Design

To map the literature available on societal implications of EUCS, we decided to conduct a scoping review. A scoping review instead of a systematic review was chosen because scoping reviews are more exploratory in nature, allow for a broader review of literature available with more expansive inclusion criteria and may provide directions for future research [17]. In conducting this review, we adhered to the Preferred Reporting Items for Systematic Reviews and Meta-analyses extension for Scoping Reviews (PRISMA-ScR) checklist [18].

### Search strategy

To perform our search, we used the citation-based method CoCites [19]. CoCites uses previously identified highly relevant scientific publications that fulfill the inclusion criteria (*query articles*) and performs two searches: (1) a co-citation search that retrieves the reference list of the query article and identifies which articles in this list are most often cited together with it, and (2) a citation search that finds all articles that cite or are cited by the query article. For a single query article, the citation search retrieves its citations and references. For a set of related query articles, the citation search finds articles that cite or are cited by multiple articles from the query article set. This step is performed to include articles that are recently published but not (yet) cited frequently enough to appear in the co-citation search [19]. CoCites thus differs from the traditional review approach in that it starts with identification of one or more highly relevant publications and uses these publications as the basis of the search. While it is a more efficient approach to performing a search, a limitation of this approach is that CoCites may not retrieve infrequently cited publications that themselves do not cite or are not cited by any articles from the query set, therefore potentially missing very old articles or very recently published articles [19].

To perform our search, one or more query articles needed to be defined. To this aim, authors with expertise in this field (MP, IL, LH, WD) as well as an external sociologist, discussed societal implications of EUCS they hypothesized. In this discussion, societal implications were defined as consequences of EUCS for society instead of for the individual. Based on this, the experts reached consensus on a set of five definite query articles written by a blended group of authors [2, 3, 14, 20, 21]. A set of five query articles was defined as these were thought to cover all hypothetical implications defined by the experts. We then performed analyses on these five query articles using CoCites. The search was conducted in October 2020 and updated in May 2022.

### Selection of articles

**Fig. 1:**
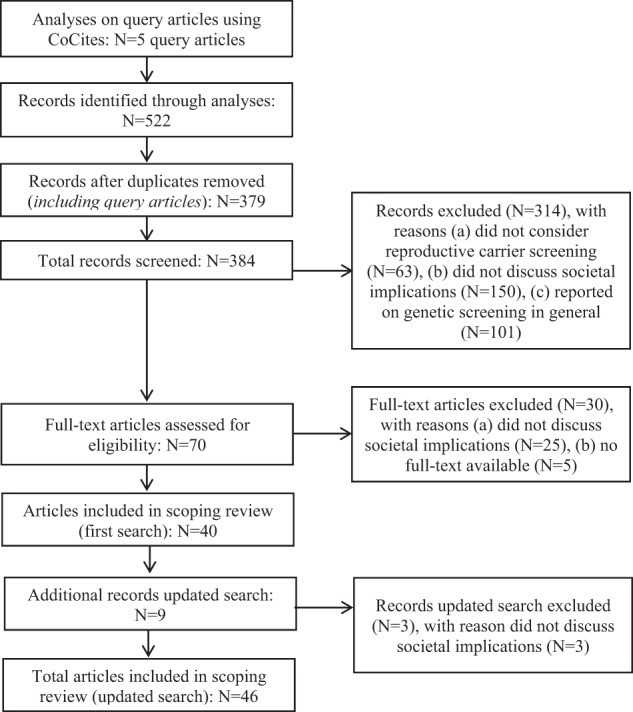
Flow-chart of the article selection process.

Figure 1 presents the flow-chart of the article selection process. Articles that were cocited more than once were included in the selection process. We included articles that: 1) discussed potential societal implications of universal offers or reported on potential and experienced societal implications of earlier ancestry-based screening offers that could shed light on the implications of an EUCS offer, and 2) were written in English. We included all design types, both articles reporting primary data as well as expert consensus papers, opinion papers and literature reviews, as we aimed to explore all types of data and insights available on (potential) societal implications of EUCS knowing that few primary data on ECS in particular would be available.

Titles and abstracts were screened for eligibility by two of the authors (LvdH and NvdB) independently, using the software program Rayyan [22]. Subsequently, full texts of the remaining articles were screened and read by LvdH and NvdB to select articles for inclusion. Any discrepancies between authors during the selection process were discussed until consensus was reached. In case of disagreement, a third author (MP) was available to reach agreement. Inter-rater reliability was assessed as high (Κ = 0.82) [23].

### Data synthesis

Data synthesis was performed independently by two of the authors (LvdH and NvdB). We used a narrative synthesis strategy to summarize (potential) societal implications of an (expanded) carrier screening offer [24]. For each article, data were extracted, analyzed, and coded using a data extraction form. A codebook was based on the societal topics discussed by the authors prior to the search and was further developed based on data synthesis. As this was a scoping review, we performed no quality assessment on the included articles.

## Results

### Study characteristics

Our search yielded 522 articles (see Fig. 1). After removing duplicates, 379 articles remained, including the five query articles. Seventy articles were selected for full-text analysis after title and abstract screening, and we eventually included 46 articles in this review. Articles were excluded when there were no societal implications discussed (*N* = 28) or when no full-text was available (*N* = 5). Table 1 gives an oversight of the main characteristics of the articles included. Authors were from the United States and Canada [25–32], Australia and New Zealand [13, 33, 34], the United Kingdom [35, 36], the Netherlands, [2, 5, 20, 37–49] Sweden, [14, 50–53] Belgium [10, 54], Israel, [6, 55–57] France [58] and Cyprus [4, 7], or consisted of a panel of European or international stakeholders [3, 21]. Most articles were published after 2011. [2, 3, 5, 9, 10, 13, 14, 20, 21, 25–31, 33, 35, 36, 38–41, 43–55, 58] Multiple article types were present in this selection, including primary data of quantitative survey studies [30, 38, 44, 48, 50, 58] and qualitative studies, [2, 6, 29, 39, 46, 49, 51–55] a witness seminar [41], a workshop with experts [3], descriptions of previous screening offers or evaluations thereof [21, 40, 42, 43, 57], theoretical papers [4, 7, 13, 14, 20, 27, 28, 31, 32, 34, 35, 45, 47], literature reviews [10, 33, 36, 56], an expert consensus paper [5], cost-effectiveness analyses [25, 26] and a sociotechnical analysis [37].

**Table 1:** Characteristics of articles included.

First author	Year	Region	Article type	Aim of article	Screening focus	Main conclusions
Wilfond [32]	1990	US	Theoretical paper	To review past American experiences and problems with mass screening programs and explore the potential for similar problems with a program for cystic fibrosis.	Cystic fibrosis	The author states that screening programs should be designed to minimize error, confusion, stigmatization and discrimination and maximize the transfer of understandable information. Benefits and risks should be investigated in pilot studies.
Achterbergh [37]	2007	Netherlands	Sociotechnical analysis	To get insight into the process of implementation of a preconception screening program to identify carrier couples for cystic fibrosis and hemoglobinopathies.	Cystic fibrosis & haemoglobinopathies	The authors conclude that successful implementation of preconceptional carrier screening will have to include the establishment of a new preconceptional healthcare setting and a visible public health authority that can coordinate, monitor and evaluate the initiative.
Kalokairinou [7]	2008	Cyprus	Case description	To describe the preconception screening program for the prevention of β-thalassemia in Cyprus.	ß-Thalassemia	The author concludes that, although the PCS program in Cyprus proved a successful model for the management of ß-thalassemia, the program bears no resemblance to eugenics.
Raz [6]	2008	Israel	Qualitative interview study with ultra-orthodox women and matchmakers (*N* = 24)	To explore the views of members of the ultra-orthodox Jewish community regarding premarital carrier matching in the Dor Yeshorim program.	Expanded	This study indicates that participants in the Dor Yeshorim program have many misunderstandings regarding the genetic basis of carrier matching and that the experiences differ from the original design and public messages of the program. While Dor Yeshorim aimed to reduce individual stigmatization, the program also reproduces stigmatization by a culture of ignorance and fear.
Lakeman [42]	2008	Netherlands	Screening offer evaluation using quantitative surveys among participants originating from Europe, North America or Australia (*N* = 97) and participants originating from other parts of the world (*N* = 46) before, shortly after and three months after receiving screening results	To study psychological outcomes, knowledge, recall and understanding of test results, satisfaction, and reproductive intentions of participants in preconceptional carrier screening.	Cystic fibrosis & hemoglobin-pathies	No major adverse psychological or social effects were found. Participants participating in the carrier screening offer did not report major feelings of discrimination or stigmatization. No differences were found between the study groups in this regard.
Zlotogora [56]	2009	Israel	Literature review	To provide an overview of the advantages and disadvantages of population genetic screening programs.	Thalassemia, spinocerebellar ataxia, Tay-Sachs disease	Both mandatory and voluntary screening programs exist worldwide. ﻿For voluntary programs, a major problem is that in many cases awareness about the existence of screening tests is very sparse.
Zlotogora [57]	2009	Israel	Program description	To describe Israel’s national genetic screening program, based on the experience of the first five years.	Thalassemia & Tay-Sachs disease	The author concludes that the community program directed toward couples in their reproductive period does not seem to have led to stigmatization at either the individual or the community level.
Cowan [4]	2009	Cyprus	Program description	To describe the development of the quasi-mandated screening program for ﻿ß-thalassemia in Cyprus.	ß-Thalassemia	The author argues that the Cypriot program does not have a eugenic aim as it involves mandated premarital carrier screening combined with voluntary prenatal diagnosis and voluntary termination of afflicted pregnancies.
Modra [34]	2010	Australia	Theoretical paper	To examine how cystic fibrosis carrier screening should be offered if Australia were to initiate a universal carrier screening program.	Cystic fibrosis	The authors state that offering carrier testing before pregnancy is preferable given the ethical advantages, while screening during pregnancy should also be offered. Establishing the screening program in a non-clinical setting is recommended to reach the preconception population.
Frumkin [55]	2011	Israel	Qualitative interview study with modern-religious Ashkenazi Jewish adults (*N* = 23)	To report on attitudes of modern-religious Ashkenazi Jewish adults in Israel toward anonymous carrier matching by Dor Yeshorim and to examine decision-making about carrier screening.	Small	The findings show a significant gap between the modern-religious respondents’ attitudes and family planning strategies and the method of confidential carrier matching.
De Wert [20]^b^	2012	Netherlands	Theoretical paper	To review ethical issues of both individual preconception genetic counseling and of systematically offered PCS to couples or individuals in the reproductive age.	Small & expanded	The authors state there are moral reasons for regarding enhancement of reproductive autonomy as preferable to prevention as the primary objective of preconception carrier screening. In case of avoiding serious suffering, prevention may be a morally acceptable objective of preconception carrier screening. If preconception carrier screening is offered for cost reduction, reproductive freedom is under threat, possibly leading to pressure to also avoid the birth of children with minor or treatable conditions.
Jans [41]	2012	Netherlands	Sociotechnical analysis & qualitative witness seminar with stakeholders, including clinicians, scientists and policymakers (*N* = 14)	To explore the decision-making process of the past concerning preconceptional and prenatal carrier screening for hemoglobinopathies.	Hemoglobino-pathies	Based on witness reports, screening in general never appeared high on the policy agenda. Participants shared that registration of ethnicity remains sensitive due to the current political climate. Complexities related to carrier screening are a challenge in Dutch healthcare.
Ready [30]	2012	US	Quantitative survey study women’s healthcare providers (*N* = 203)	To determine women’s HCPs’ knowledge and attitudes regarding genetic ECS.	Expanded	Participants generally had good knowledge and positive attitudes about genetic conditions and expanded genetic screening. Very few participants disagreed with the notion of carrier screening as socially responsible behavior.
Aspinall [35]	2013	UK	Theoretical paper	To evaluate the appropriateness, operationalization and efficacy of decision instruments that utilize origins-based concepts of race, ethnicity, or ancestry to determine genetic carrier screening status.	Small & expanded	Several potential deleterious consequences of using origins-based concepts can be identified. Any such usage to describe individuals as high risk by virtue of their group membership risks potential offense, stigmatization and discrimination.
McGowan [29]	2013	US	Qualitative focus group study (*N* = 6 groups) with 40 medical geneticists and genetic counselors	To describe genetics professionals’ perceptions of the benefits and challenges of expanding prenatal carrier screening and to situate these in the social debates about integration of new risk assessment technologies into prenatal care.	Expanded	Participants believed that clinicians and pregnant women will likely face logistical and psychosocial challenges similar to those that have arisen in the context of universal carrier screening for single gene disorders and other forms of prenatal screening.
Massie [33]	2014	Australia and New Zealand	Literature review	To describe prenatal and preconception population carrier screening for cystic fibrosis in Australia and to consider progress toward establishing a universal program.	Cystic fibrosis	Literature identified showed support for prenatal and preconception cystic fibrosis carrier screening by the community, health professionals and relevant professional bodies in Australia. The barriers to development of a national screening program could be overcome with more physician engagement and government support.
Rose [31]	2015	US	Theoretical paper	To reflect on the ethical implications of ECS.	Expanded	The author argued that it is reasonable to offer ECS to all patients. ECS should remain voluntary in any form and requires informed pre-test counseling, independently derived guidelines and oversight from professional organizations.
Langlois [28]	2015	Canada/US	Theoretical paper	To describe an authors’ debate on whether preconception ECS should replace current prenatal screening for specific gene disorders.	Expanded	The authors argue that although preconception ECS maximizes reproductive options for at-risk couples, prenatal screening would need to remain an integral part of prenatal care as long as there is a significant proportion of pregnant women who do not receive regular medical care prior to pregnancy.
Kihlbom [14]^b^	2016	Sweden	Theoretical paper	To provide a conceptual framework that may enhance ethical debate of preconception care in general and (preconception) ECS specifically.	Expanded	A universal offer of (preconception) ECS raises several ethical issues around justice (including prioritization, discrimination and stigmatization), consequences (including medicalization) and autonomy (including informed consent and routinization). The author also argues that it is ethically problematic to have a discrepancy between an official aim of ECS offers and what really drives development.
Van der Hout [2]^b^	2016	Netherlands	Qualitative interview study with Dutch key stakeholders working in the field of carrier screening (*N* = 17)^c^	To gain insight into professionals’ opinion on EUCS and to provide a balanced picture of the potential pros and cons of EUCS.	Expanded	Stakeholders perceived potential benefits of EUCS offers. However, they also had numerous concerns. The findings of this study indicate that if certain concerns do not receive proper attention, EUCS may do more harm than good.
Henneman [5]	2016	Europe	Expert consensus paper, with members of the Public and Professional Policy Committee of the European Society of Human Genetics	To contribute to the public and professional discussion regarding responsible implementation of ECS and to arrive at better clinical and laboratory practice guidelines.	Expanded	The experts advise that to minimize potential adverse psychological and social implications and ensure the quality of tests, carrier screening tests should comply with quality control and information standards. They also state that research into the financial aspects of ECS into public health systems is needed. It is recommended that the primary purpose of ECS should be to enhance reproductive autonomy, there should be sufficient evidence before ECS is initiated, it should ideally be offered preconceptionally, that psychosocial support and genetic counseling is provided, that the model for reaching informed consent is evaluated, that participation in ECS should be voluntary and informed, that HCPs should be educated and trained and governments and that public health authorities should adopt an active role in discussing responsible ECS implementation.
Azimi [25]	2016	US	Cost-effectiveness analysis based on published literature, population surveys and expert opinion	To evaluate the cost-effectiveness of carrier screening using NGS versus genotyping for 14 of the recessive disorders for which medical society guidelines recommend screening.	Expanded	The findings of this study indicate that NGS-based ECS offers in multi-ethnic populations show greater benefit in clinical outcomes and lower total healthcare costs compared to current genotyping-based carrier screening, although these findings are sensitive to assumptions with regard to mutation detection rates and carrier frequencies in multi-ethnic populations.
Holtkamp [38]	2016	Netherlands	Quantitative survey study with individuals from the Dutch Jewish community (*N* = 145)	To explore attitudes of people from the Jewish community regarding ancestry-based and pan-ethnic ECS.	Small & expanded	Slightly more than half of the respondents favored EUCS over ancestry-based screening. Important reasons in favor of a EUCS were that everyone has a right to be tested, the fear of stigmatization when offering ancestry-based panels and difficulties in identifying risk based on mixed backgrounds. Prevention of high healthcare costs was considered the most important reason against EUCS.
Ekstrand Ragnar [50]	2016	Sweden	Quantitative survey study with expecting couples (*N* = 777)	To investigate parents’ (future) interest and motives toward preconception ECS and factors associated with interest in preconception ECS.	Expanded	Most participants expressed uncertainty about whether they would consider preconception ECS in the future, but women more often expressed motives against preconception ECS, including being concerned about negative consequences and being opposed to such a way of child selection.
Matar [53]	2016	Sweden	Qualitative interview study with HCPs (*N* = 11), including clinicians, geneticists, a midwife, and a genetic counselor	To explore Swedish HCPs’ views on preconception ECS.	Expanded	Participants raised concerns about ethical issues including medicalization, reproductive autonomy, parental responsibility, discrimination against those with disease or carrier status, prioritization of health resources and uncertainty concerning what to test for and how to interpret the results.
Holtkamp [39]	2017	Netherlands	Qualitative interview study with Dutch key stakeholders working in the practical and scientific field of carrier screening (*N* = 17)^c^	To identify general and population-specific barriers and needs reflected by stakeholders regarding the implementation of ECS in a changing landscape.	Expanded	Barriers were identified on cultural level (including uncertainty about the desirability of screening and lack of priority), on a structural level (including need for guidelines, financial structure, and practical tools for overcoming challenges during counseling and need to train and educate HCPs) and practical barriers (including lack of demand for screening by the public and need for a division of responsibilities).
Molster [3]^b^	2017	International	International workshop with experts (*N* = 41)	To identify key public policy issues related to preconception ECS that governments should consider when deciding whether to publicly fund such programs.	Expanded	Participants raised a range of issues that require careful examination prior to implementing ECS. Based on the workshop outcomes, new criteria need to be developed that inform whether to initiate preconception ECS. Participants felt it was needed to consider equitable availability and downstream effects on and costs of follow-up interventions for those identified as carriers.
Holtkamp [40]	2017	Netherlands	Literature review & case descriptions	To identify critical factors involved in successful implementation of ECS from a user perspective and by learning from implemented initiatives.	Expanded	This study indicates that factors contributing to successful implementation of carrier screening include familiarity with genetic diseases, its availability, high perceived benefits of screening, acceptance of reproductive options, perceived risk of being a carrier and low perceived social barriers such as stigmatization. High social cohesion in a community would also facilitate implementation.
Janssens [54]	2017	Belgium	Qualitative interview study European clinical and molecular geneticists with expertise in carrier screening (*N* = 16)	To explore geneticists’ attitudes about the implementation of ECS for monogenic recessive disorders.	Expanded	In considering ECS implementation, participants perceived benefits (including greater access to screening) but also identified major challenges, including limited benefit for most couples in the general population, lack of public and HCP education on ECS, potential negative societal implications (including slippery slope, effects for people living with conditions screened) and limited economic resources. Participants called for more extensive discussion of the societal effects of EUCS offers.
Chokoshvili [10]	2017	Belgium	Literature review	To highlight the distinct issues associated with three different types of ECS offers (direct-to-consumer, physician-mediated and clinic-based testing).	Expanded	Although all the three models (direct-to-consumer, physician-mediated and clinic-based testing) for ECS offers are characterized by different challenges, it was found that, in practice, physician-mediated ECS offers appear to be the most problematic, warranting greater regulatory scrutiny into these kind of testing offers.
Mathijssen [43]	2018	Netherlands	Screening offer evaluation using quantitative surveys among members of a Dutch founder population (*N* = 319; 182 attendees at an outpatient clinic and 137 non-attendees) and qualitative semi-structured interviews with 7/8 identified carrier couples	To evaluate experiences with a PCS offer for four recessive disorders in a Dutch founder population.	Expanded	Almost all screened individuals reported being very satisfied with screening. Genetic counseling prior to screening was preferred. Before consultation, three individuals expected to be seen differently if they turned out to be a carrier. None of the participants perceived screening as discrimination. Fear of stigmatization was not an issue for carrier couples.
Van der Hout [45]	2018	Netherlands	Theoretical paper	To provide an ethically sustainable account of the aims of EUCS.	Expanded	Enhancing autonomy appears the most appropriate aim of prenatal screening offers. However, with offers in the preconception phase, a wider range of reproductive options is available. Therefore, the authors argue that to the extent that increased control over passing on a genetic disorder raises questions of parental responsibility, it seems necessary that the account of the aims of EUCS is wider than just enhancing reproductive autonomy.
Kraft [27]	2019	US	Theoretical paper	To report on the current landscape of ECS and to identify potential challenges that should be addressed.	Expanded	The authors describe different challenges for ECS offers, including lack of demand from the public, low prioritization of health systems, potential for pressure to undergo screening, the possibility of disability-based discrimination, needed adaptations to pre- and post-test counseling, technical limitations and the evolving technological and socio-political landscape.
Beauchamp [26]	2019	US	Cost-effectiveness analysis comparing minimal screening and a 176-condition ECS panel	To model the clinical impact and cost-effectiveness of preconception ECS.	Small & Expanded	This study demonstrates that, relative to minimal screening, preconception ECS reduces the affected birth rate and is estimated to be cost-effective (<$50,000 incremental cost per life-year).
Nijmeijer [44]	2019	Netherlands	Quantitative survey study with members of the Dutch general population (*N* = 781) in the reproductive age	To assess the attitudes of the general Dutch population toward preconception ECS and to investigate which factors influence these attitudes.	Expanded	This study indicates an overall positive attitude toward ECS. 55% believed that ECS should be offered to all prospective parents. About one third of participants perceived adverse societal implications, including slippery slope, discrimination and societal pressure.
Matar [51]^a^	2019	Sweden	Qualitative interview study with Swedish healthcare policymakers (*N* = 10)	To explore how healthcare policymaking experts perceive ethical and social aspects of preconception ECS.	Expanded	Participants believed that Sweden is currently not ready to implement ECS due to several ethical and social concerns, such as risks of stigmatization and discrimination, and potential long-term effects, such as a change of the public mind-set from tolerance and societal responsibility for the disabled to intolerance and attitudes of blame.
Matar [52]^a^	2019	Sweden	Qualitative interview study with Swedish healthcare policymakers (*N* = 10)	To explore values and value conflicts that healthcare experts recounted in relation to the discussion of implementation and use of preconception ECS in Sweden.	Expanded	Participants described values and value conflicts with regard to EUCS implementation related to solidarity (including equality, justice and social care) and respect for individuals (including autonomy, integrity and privacy).
Delatycki [21]^b^	2019	International	Case descriptions	To describe reproductive carrier screening offers across the globe.	Expanded	The authors describe carrier screening programs around the world that target different groups, as does the governance structure. Differences relate to geographical variation in carrier frequencies of genetic conditions and local healthcare, financial, cultural and religious factors.
Dive [13]	2020	Australia	Theoretical paper	To reflect on the ethical implications of large scale ECS offers in Australian healthcare.	Expanded	This article describes many ethical implications of ECS offers, related to its aim, gene selection, consent, funding and reporting of results. The authors argue that future delivery of a national ECS program in Australia will need to recognize and respond to this diversity while also upholding the values that motivate the program.
Rowe [36]	2020	UK	Literature review	To evaluate the existing evidence for EUCS programs, and to appraise the potential benefits and challenges of implementation within a publicly funded healthcare system.	Expanded	Literature identified indicates that although preconception screening would maximize the potential benefits from EUCS, the resource implications of different modes of delivery need to be carefully evaluated and balanced against maximizing reproductive autonomy and ensuring equity of access.
Cornel [47]	2021	Netherlands	Theoretical paper	To demonstrate how the dynamics and expansions in NBS and carrier screening have challenged four well-known screening criteria (treatment, test, target population and program evaluation), and the decision-making based on them.	Expanded	The authors argue that that shifting perspectives on screening criteria for NBS as well as carrier screening lead to converging debates in these fields.
Conijn [46]	2021	Netherlands	Qualitative focus group study with members of the general population (*N* = 18) and relatives of MPS III patients (*N* = 23)	To obtain insight into the perspectives of relatives of MPS III patients and potential users from the general population on ECS.	Expanded	Participants, both relatives of MPS III patients and individuals from the Dutch general population, support implementation of preconception ECS to prospective parents in the general population. However, important barriers for ECS implementation were identified. The concept of EUCS showed to be difficult to understand, particularly for participants from the general population.
Van Dijke [48]	2021	Netherlands	Quantitative survey study with high-risk participants (*N* = 89) and general-risk participants (*N* = 43)	To evaluate the experiences of couples that participated in an expanded carrier screening offer.	Expanded	Most individuals participating in the carrier screening program showed to have made an informed choice, showed no or limited negative psychological impact, and were satisfied with the screening although they considered the cost of the test too high.
Dive [13]	2021	Australia	Theoretical paper	To describe the relevance of public health ethics for implementation of reproductive genetic carrier screening programs.	Expanded	The authors argue that EUCS has sufficient features in common with other public health screening programs that it becomes important also to attend to public health implications of EUCS. It is stated that not attending public health implications may affect the capacity of people to exercise their reproductive autonomy.
Morberg Jamterud [49]	2021	Netherlands	Qualitative interview article with general practitioners (*N* = 7), compared with a theoretical bioethical analysis study	To present an empirical bioethics analysis of the PECS practice from the perspective of GPs.	Expanded	Two themes were identified in the analysis: (1)‘Choice and its complexity’, which suggests that medical, moral, social and psychological aspects co-shape the complexity of choice regarding EUCS and (2) EUCS as prompting existential concerns’, indicating that it is not possible to analyse existential issues raised by EUCS solely on the level of the couple or family but that a societal level must be included, since these levels affect each other (referred to as ‘entangled existential genetics’).
Bonneau [58]	2022	France	Quantitative survey article among members of the general population (*N* = 1568)	To collect French general population opinion on the implementation of preconception genetic testing and explore the factors associated with a difference of opinion.	Expanded	In this study, 91% of participants was favorable to ECS and 57% declare to be willing to participate in screening. A medical prescription by a general practitioner or a gynecologist was considered best to offer screening for 73%, with a reimbursement from insurance. However, 19% declared not to be willing to participate because of ethic or moral convictions, and the fear that the outcome would question becoming pregnant. Otherwise, most participants considered the test medical progress despite the risk of an increased medicalization of the pregnancy.

*US* United States of America, *UK* United Kingdom, *PCS* preconception carrier screening, *ECS* expanded carrier screening, *NGS* next-generation sequencing.

^a^These articles describe different results from the same study.

^b^Co-citation analyses was performed on these five query articles.

^c^These articles describe different results from the same study.

### Themes

Three main themes regarding (potential) positive and negative societal implications were identified: (1) Medicalization, (2) Stigmatization and discrimination of carriers and of people affected with the conditions screened and (3) Challenges in achieving equitable access. Table 2 presents the themes addressed per article included in this review.

**Table 2:** Themes and subthemes on societal implications of EUCS discussed per article.

Reference	Medicalization			Stigmatization & discrimination			Challenges on achieving equal access		
	Fear of supply push	Fear of routini-zation	Slippery slope	Expectations regarding stigmatization	Expectations regarding discrimination	Experiences with occurrence stigmatization and discrimination	Access of ancestry-based vs. pan-ethnic screening	Reaching the target population	Costs and funding
Wilfond [32]		+		+		+		+	+
Achterbergh [37]								+	
Kalokairinou [7]						+			+
Raz [6]		+				+			
Lakeman [42]						+			
Zlotogora [56]		+							
Zlotogora [57]						+			
Cowan [4]			+						
Modra [34]		+						+	
Frumkin [55]		+				+			
De Wert [20]				+	+				
Jans [41]				+					
Ready [30]		+			+				
Aspinall [35]				+					
McGowan [29]		+	+	+				+	
Massie [33]							+		
Rose [31]								+	+
Langlois [28]				+			+		
Kihlbom [14]	+	+	+	+	+				
Van der Hout [2]	+	+		+	+		+	+	
Henneman [5]				+	+	+	+	+	+
Azimi [25]									+
Holtkamp [38]		+		+			+	+	+
Ekstrand Ragnar [50]	+								
Matar [53]		+	+	+	+				+
Holtkamp [39]				+				+	+
Molster [3]		+	+	+	+				+
Holtkamp [40]									
Janssens [54]			+	+			+		+
Chokoshvili [10]									+
Mathijssen [43]						+			
Van der Hout [45]		+		+			+		
Kraft [27]		+		+	+			+	+
Beauchamp [26]									+
Nijmeijer [44]	+	+					+		+
Matar [51]		+	+	+					+
Matar [52]		+	+	+	+			+	+
Delatycki [21]				+	+	+			+
Dive [13]		+		+					+
Rowe [36]		+		+				+	+
Cornel [47]				+	+			+	+
Conijn [46]	+		+						+
Van Dijke [48]									+
Dive [13]		+		+				+	+
Morberg Jamterud [49]		+							+
Bonneau [58]				+	+				

### Potential societal implications related to medicalization

Medicalization is a term used to describe potential unwanted effects of implementing medical procedures on aspects of life that were previously understood as being outside the realm of healthcare [14]. Several articles discussed aspects of medicalization as potential negative societal implications of EUCS (Table 2). In most articles, medicalization was by authors or stakeholders hypothesized as a negative societal implication of EUCS. Empirical evidence that such implications occur is however scarce (Table 3). The potential negative effects related to medicalization listed were: (A) supply push of EUCS [2, 14, 39, 40, 44, 46, 50], (B) routinization of EUCS or its uncritical use [2, 3, 6, 13, 14, 27, 29, 30, 32, 34, 36, 38, 44, 45, 49, 51–53, 55, 56] and (C) that EUCS may contribute to a societal climate where having less than ‘perfect children’ is regarded as problematic. [3, 4, 14, 29, 46, 51–54] These subthemes are described in more detail below.

**Table 3:** (Potential) negative and positive societal implications of EUCS identified in review.

(Potential) negative implications	Empirical evidence found in search^a^
*Medicalization*	
Supply push	No evidence found in search
Routinization	Evidence for [55]
Slippery slope	No evidence found in search
*Stigmatization*	
Reinforcement of disability-based stigmatization	No evidence found in search
Stigmatization of carriers/couples opting out of screening	Evidence for [5–7, 21, 32, 55] Evidence against [42, 43, 57]
*Discrimination*	
Discrimination of carrier couples	Evidence for [5, 21, 32] Evidence against [42, 43, 57]
Discrimination of people living with the conditions screened	No evidence found in search
*Achieving equal access*	
Awareness high-risk groups undermined by universal offer	No evidence found in search
Reaching target population (unplanned pregnancies, inadequate information provision)	No evidence found in search
Costs & funding: couples perceived barriers regarding paying for screening	Evidence for [21, 48]
**(Potential) positive implications**	
*Stigmatization*	
Reduce stigmatization of ethnic groups	No evidence found in search
*Achieving equal access*	
Equal access for high-risk groups and the general population	No evidence found in search
**Other**	
*Achieving equal access*	
Costs & funding: cost-effectiveness/fair allocation of healthcare resources	No evidence found in search

*EUCS* expanded universal carrier screening.

^a^Empirical evidence for societal implications was only found in relation to high-risk offers (often for one or few conditions), except for the study of Van Dijke et al. [48].

### Supply push

The term ‘supply push’ was used by Bekker et al. [59] in the context of cystic fibrosis carrier testing, where the authors showed that the strongest variable associated with screening uptake was the (opportunistic) approach by healthcare professionals (HCPs) and suggested that high uptake rates more likely represent the active carrier screening approach initiated by HCPs or government rather than a response to a current demand from the general public. In the interview study of Van der Hout et al. [2], Dutch stakeholders additionally suggested that implementing EUCS as a top-down initiative prompted by the healthcare system is a matter of supply push. As a consequence, they stated that the general public might lack the initial desire to know their carrier status, either because a frame of reference is lacking or because the information EUCS provides does not respond to a problem recognized in daily practice, e.g. due to lack of awareness [2]. This was also observed in a qualitative stakeholder study with potential users from the general public, indicating that they were not aware of the availability of EUCS and therefore did not consider to participate yet [46]. Kihlbom [14] stated that resistance to partaking of EUCS may exist as well, as couples may oppose the idea of implementing an additional medical procedure in the preconception or prenatal phase. Additionally, people with certain religious beliefs or other world views may have less interest in screening that may allow for selective reproduction [44, 46, 50]. Prior experience or familiarity with the condition(s) screened would lead to a higher expressed need for EUCS [39, 40].

### Routinization

Several articles mentioned concerns regarding routinization: when screening becomes routine, as could happen when offered programmatically, it may in the end be offered and accepted without careful consideration [60]. Routinization could be worrisome for an EUCS offer as part of a reproductive screening program because this may affect informed decision-making and the freedom to choose, both key values in reproductive carrier screening, and may have unwanted consequences for people with disabilities [61].

Theoretical articles included in this review mentioned that if a universal offer becomes routine, couples may believe that partaking in EUCS is the right thing to do and may even lead to societal pressure on couples to participate in testing [3, 13, 14, 27, 34, 36]. Articles stated that routinization of EUCS may specifically affect societal perspectives on reproductive responsibilities, raising the question whether couples will feel free to decline the offer or to make the decision not to intervene to prevent an affected pregnancy if they appear to be a carrier couple [6, 13, 14, 27, 32, 36, 45]. This concern was also expressed by HCPs and policy makers. [2, 29, 49, 51–53] Stakeholders moreover stated that if EUCS becomes routine, it may impact the perception of health in the sense that risks need to be prevented [29, 52]. Ready et al. [30] reported that 33% of women’s HCPs were ambivalent regarding the notion that carrier screening is socially responsible behavior. In a study exploring attitudes of the general public, 44% agreed that EUCS creates high expectations of parents to conceive a healthy child [44].

In addition, some authors argued that the organization of such an offer may reinforce the impact of routinization [3, 13, 32, 34]. In a theoretical articles it was stated that offering ECS in a clinical setting may lead couples to compliantly accept screening, as they may believe screening is necessary or routine when clinically offered [13, 34]. Authors additionally stated that government-sponsored ECS programs, full reimbursement of ECS, using an easy and relatively non-invasive manner for sample collection, and public campaigns about the possibility of ECS may foster the notion of ECS being routine and that it is desirable to partake and perhaps even to reduce the conditions screened [3, 13, 32]. A survey study among Dutch Jewish participants showed that 24.1% believed that HCPs were allowed to insist that Jewish people participate in screening [38].

One article actually presented empirical data on routinization and subsequent societal pressure in case of a (semi-mandatory or mandatory) ancestry-based carrier screening offer in Israel [55]. This study on attitudes of modern-religious Ashkenazi Jewish people in Israel found that most respondents confirmed that they succumbed to societal pressure when they took part in the premarital screening program [55].

### Aim for ‘perfect children’

Several articles included in this review also suggested that EUCS may contribute to a societal climate where having less than ‘perfect children’ is regarded as problematic, especially when mild conditions would be included in the test panel(s). These articles included theoretical papers [14] and stakeholder consultations. [3, 29, 46, 51–54] Related to this, some policy makers in Sweden expected that members of the general population might misconstrue the government’s motives for ECS, considering past experiences with state-promoted eugenics policies [51]. Other stakeholders additionally shared concerns that a universal offer may indeed reinforce notions of the government restricting reproductive freedom [3, 53]. However, Cowan et al. [4] argued that the scenario of new eugenics policies occurring in Western democratic societies was considered implausible, as the considered aim of the program is to enhance reproductive autonomy at the individual level rather than to improve the health or wellbeing at population level. No articles reported experiences with actual carrier screening offers that are relevant for assessing the reality of these concerns.

### Stigmatization and discrimination

Multiple aspects of genetic stigmatization (i.e. labeling a group or person with negative medical, social or psychological characteristics) and discrimination (i.e., making unjustified distinctions between groups or persons because of medical, social or psychological characteristics [62]) were brought forward as potential implications of a ECS offer [2, 3, 5, 13, 14, 20, 21, 27–30, 32, 35, 36, 38, 39, 41, 45, 47, 51–54, 58] or were reported as effects of ancestry-based preconception screening offers of the past [5, 7, 21, 32, 42, 43, 55, 57]. Both potential positive and negative societal implications related to stigmatization and discrimination were described in theoretical articles in the context of a universal offer, as outlined below. We found no empirical evidence of stigmatization and discrimination following EUCS.

### Expectations regarding stigmatization

As EUCS will be offered to all individuals in the general population regardless of ancestry, consanguinity or geographic origin, stakeholders [38, 39, 58], experts and authors [5, 21, 28, 35, 36, 45, 47] stated that such an offer could potentially reduce stigmatization of ethnic groups. Stakeholders additionally suggested that a universal offer may normalize carrier status, because ‘we are all fellow mutants together’ (p.169) [63] regarding carrier status for recessive conditions [29].

However, potential harms of EUCS regarding stigmatization were mentioned in multiple articles as well. First, stakeholders believed that the offer might reinforce disability-based stigmatization, [2, 41, 51–54] which was also mentioned in theoretical papers [13, 14, 20, 27, 45] and an expert consensus paper [5]. They suggested that stigmatization may occur because the offer might encourage the perception that there is no place for people with disabilities in our society, or that the lives of people with genetic conditions have less value, referred to as ‘the disability rights critique’. [2, 5, 13, 14, 27, 32, 45, 51–54] Some authors [13, 27] argued that the risk of increased stigmatization depends on the screening panel, stating that it would be more ethically defensible to screen for genetic variants that cause severe childhood-onset conditions, although perceptions on severity are not entirely objectifiable.

Second, stakeholders reflected upon potential stigmatization of carriers [3, 53] as well as those who opt not to undergo screening, particularly when it results in affected children [2, 51, 53]. This was also stated in theoretical papers [14, 20, 27, 36]. In a study among Swedish stakeholders, it was stated that if partaking in screening may be considered a couple’s reproductive responsibility, blame and guilt may become associated with declining the offer, especially when a child is affected [51]. These Swedish stakeholders [51], as well as an author [32], additionally mentioned that for carriers or those opting out of screening, it may become challenging to find a partner. Furthermore, stakeholders [2, 51] and authors [20] mentioned that stigmatization of affected people may lead to increased societal pressure to partake in screening.

### Expectations regarding discrimination

In addition, stakeholders mentioned that an EUCS offer could lead to discrimination against carrier couples or may affect wellbeing of people living with the conditions screened for [2, 3, 47, 52, 53]. Some theoretical and expert articles [3, 5, 14, 20, 21, 47] suggested that a universal offer might lower the incidence of affected births, which, in turn, could lower the incentive to develop treatments, thus disadvantaging people living with the conditions screened for. It was also expected that the societal willingness to support families with affected children could decrease as a result [2, 47]. Other HCPs were worried about increases in insurance rates as well [30]. These concerns were also expressed in a theoretical article [27]. About half of French participants from the general population expressed fear of being discriminated if they were identified as a carrier [58].

### Experiences with stigmatization and discrimination in carrier screening offers

None of the articles reported empirical evidence regarding stigmatization and discrimination as a consequence of EUCS offers (Table 3). Multiple studies on high-risk carrier screening offers however reported on participants’ experiences with aspects of stigmatization and discrimination. In the 1970s, ancestry-based sickle cell disease screening in the United States led to increased stigmatization and discrimination of carriers in access to employment and education and in the ability to obtain life insurance [5, 21, 32]. Stigmatization of carriers was additionally reported as a consequence of the Jewish orthodox premarital screening offer [6, 55] and as a result of the semi-mandated screening offer for beta-thalassemia on Cyprus [7]. A positive carrier status resulted in restricted opportunities to marry, especially for families with affected family members [6, 7, 55]. In contrast, in studies reporting on ancestry-based preconception screening in the Netherlands, couples did not experience stigmatization or discrimination [42, 43]. No program-related stigmatization was found in another study evaluating a preconception screening offer in Israel [57]. Participants from Israel in a study of Raz et al. [6] felt that acceptance of people with disabilities in their community increased.

### Challenges in achieving equitable access

Several articles suggested potential societal implications of a EUCS offer related to equitable access, including: (A) consequences for access of a high-risk only versus a universal offer [2, 5, 28, 33, 38, 44, 45, 54], (B) challenges in reaching the target population [2, 5, 13, 27, 29, 31, 32, 34, 36, 37, 39, 47, 52] and (C) potential implications related to costs and funding. [3, 5, 7, 10, 13, 21, 25–27, 31, 32, 36, 38, 39, 44, 46–48, 51–54] These are outlined below. Only one article reported empirical evidence for actual implications of funding for access to carrier screening (Table 3) [21].

### Equitable access in a high-risk only versus a universal offer

Multiple articles mentioned that, as it becomes increasingly difficult to assign single ethnicities to people in today’s increasingly multi-cultural societies, targeted screening offers are insufficient. They argued that it is reasonable to offer ECS universally, regardless of race or ethnicity, to promote equitable access to screening [5, 28, 45]. In this context, HCPs mentioned that an advantage of EUCS is that it promotes equitable access as it is offered to all couples with a desire to have children [2, 54]. A participant in the stakeholder interview study of Van der Hout et al. [2]. even argued that it would be unethical to deprive couples of such an offer. This was indicated by Dutch studies reporting on the perspectives of at-risk communities as well [38, 42, 43] and members of the general population [44], indicating a preference for carrier screening to be available to everyone. On the other hand, experts suggested that awareness amongst at-risk groups, who benefit most from ECS, may be undermined by a universal offer [5]. Therefore, Dutch stakeholders believed that EUCS should not completely replace high-risk offers [2]. We found no articles presenting data on experiences regarding this subtheme in actual carrier screening offers.

### Challenges in reaching the target population

Furthermore, articles suggested that reaching the target population preconceptionally might be difficult. Some articles discussed for example that, as not every pregnancy will be planned or couples do not visit HCPs before pregnancy, these individuals or couples will not receive the screening offer if limited to the preconception phase [20, 34, 36, 37]. Cornel et al. [47]. additionally mentioned that preconception care is not systematically offered, also limiting the feasibility of offering EUCS in the preconception phase. In addition, several articles, including stakeholder consultations and theoretical papers, raised a concern about whether informed (autonomous) decision-making could actually be reached. They mentioned that successfully transferring the information on ECS to everyone may be complicated, specifically in couples with lower health literacy or educational level [5, 27, 29, 31, 32, 34, 36, 39, 52]. Some stakeholders specifically raised concerns about the knowledge and awareness in the general population, but also amongst HCPs, about genetics, screening, and reproductive options, possibly complicating informed decision-making [39]. These concerns were expressed in the theoretical article by Wilfond et al. [32] as well. Stakeholders additionally wondered whether reaching informed consent would become more complicated with larger panels of conditions [2, 29]. Modra et al. [34] voiced the worry that it may specifically be complicated to reach informed decision-making in case of EUCS in a prenatal setting, because of the time pressure on making a decision. Finally, authors of a theoretical article mentioned that accessibility of EUCS in regional, rural and remote areas as well as access of culturally and linguistically diverse communities is also a point of concern [13]. None of the articles reported carrier screening participants’ experiences on this subtheme.

### Costs & funding of service

Other challenges in relation to equitable access had to do with costs and funding. First, several articles reported on potential societal implications of couples paying a fee for screening [5, 13, 27], including stakeholders. [38, 46, 52–54] Some articles, both theoretical articles [13, 32, 36] and stakeholder articles [38, 39, 44, 46], suggested that a reimbursed offer may minimize inequity and discrimination based on socioeconomic status, as compared to commercial offers, and that paying a fee for screening could potentially increase them. Relatives of patients with a severe recessive condition however mentioned that costs of screening were highly favorable compared to the costs of caring for a child affected [46]. Other stakeholders argued for partial financing, in which prospective parents would pay a certain amount for ECS themselves [39, 51, 54]. Some articles described funding strategies of current carrier screening programs. In the United Kingdom, carrier testing is offered free of charge as part of National Health Services [21]. In Israel, carrier testing is free of charge as well, and only a small fee is asked for additional testing for less frequent or severe conditions [21]. Only one article evaluated the costs of EUCS in an empirical study, with couples paying 650 euro for participating in screening, indicating that almost half of participants believed the costs of screening were too high [48]. Some of participants who were interviewed also believed that the costs could lead to inequality in access [48]. One article reported on funding and participation in an ancestry-based offer: Tay-Sachs disease screening in Australian high schools has been offered free of charge with a participation rate of 96% [21].

Second, multiple articles stated concerns whether healthcare resources will be fairly allocated in case of an EUCS offer. [10, 13, 25–27, 31, 36, 47, 49] Many stakeholders believed EUCS to be expensive and considered it not to be a priority in healthcare [33, 39, 51, 53]. Other authors suggested that an offer of EUCS should not be competing with resources to provide treatment of people affected by the conditions screened [3]. Theoretical articles stated that EUCS would increase costs, as compared to a high-risk offer only [31, 36], and HCPs in another article expressed concerns that EUCS implementation may negatively affect the budgets of other healthcare areas [53]. Compared to minimal or no screening, multiple articles expressed the expectation that EUCS would reduce the medical expenses for lifetime care of affected patients, which may lead to cost-effectiveness [25, 26, 51]. This was the case for beta-thalassemia screening in Cyprus [7]. It was also argued that in communities with a relatively high frequency of affected patients limiting (healthcare) budgets, societal impact of these diseases can be considered an argument pro EUCS [20, 47]. Saving costs for society by preventing the birth of a severely affected child was also mentioned by potential users in a qualitative stakeholder analysis study [46]. However, it was additionally argued that assessing cost-effectiveness is irrelevant in case of EUCS, as increased autonomy rather than reducing birth frequency is the primary goal of the offer [13, 27, 36].

## Discussion

In this scoping review, we aimed to assess the potential societal implications of implementing EUCS described in literature. These implications were derived from publications focusing on the expected implications of offering EUCS and from relevant publications describing the theoretical as well as proven implications of non-expanded and/or ethnicity or ancestry-based carrier screening programs.

The articles identified in this review map potential positive societal implications of EUCS, including equitable access for all couples planning to conceive, normalization of carrier status (and thus reduced risk of stigmatization and discrimination) and that EUCS potentially caters to a latent need or desire of individuals or couples unaware of their reproductive risk. This review also indicates concerns about potential negative societal implications of EUCS, including routinization of reproductive screening that potentially affects freedom of choice; EUCS contributing to a society striving for the ‘perfect child’; stigmatization and discrimination of couples who opt out of screening and of carriers and individuals affected by conditions screened; and challenges in promoting equitable access to screening.

Importantly, we found little or no empirical evidence for both positive and negative potential implications, neither in literature on EUCS, nor in most publications on screening programs for at-risk populations (focusing on one or a few diseases). Empirical studies on EUCS are lacking, whilst insight into societal implications is important for implementation decisions. As outlined by Richardson et al. [15], defining outcome measures for studies evaluating EUCS programs is essential for proper evaluation of such programs as well for collecting empirical data. Such outcome measures should include these societal implications [15]. The few articles reporting evidence for the actual occurrence of societal implications in these settings primarily described experiences with stigmatization and discrimination. It has been stated that one way to minimize stigmatization and discrimination is to improve public knowledge by, e.g., public health campaigns, and to provide proper information and counseling [5]. In addition, as cultural and social stigmas play a role in shaping disease perception, it is important that EUCS programs are implemented in a cultural-sensitive manner [64].

To implement EUCS in a socially responsible manner, it is important to consider the likelihood and impact of potential negative societal implications versus the desirability of positive societal consequences. Importantly, whether certain societal implications of EUCS will actually occur is probably dependent on the way(s) EUCS is implemented, which is dependent on the expressed aim(s) of such a program [20, 45]. If EUCS is implemented with the aim of prevention only instead of reproductive autonomy, societal pressure to partake in EUCS or to choose reproductive options preventing the birth of an affected child might be higher, as might stigmatization and discrimination [13, 20, 45]. To limit the likelihood of negative societal implications occurring, it is important that reproductive autonomy, and thus the optional character of the offer, is clearly expressed when implementing EUCS, both with respect to partaking in screening as well as regarding reproductive options in case of carriership [5].

Despite reproductive autonomy being considered the primary aim of EUCS in most literature, articles in this review suggest that a routinized offer could reinforce the idea that it is logical or a right thing to do, with society possibly expecting couples to partake in screening or to make particular choices if they appear to be carriers. Furthermore, articles suggest that EUCS set-up may affect if, and to what extent, potential negative societal implications will occur. For example, while clinical or government-initiated offers may promote responsible implementation, including equitable access to screening, this review mapped concerns that such offers may also reinforce the notion that partaking in screening is the right thing to do, and therefore may contribute to societal pressure. Healthcare professionals offering counselling may even perpetuate this when this is not addressed in counselling. It is therefore important that healthcare professionals offering screening are aware of this and discuss the notions couples may have, so that they can adequately support informed decision-making and minimize the adverse impact of routinisation.

In addition, this review indicated the possibility that EUCS would be initiated top-down rather than bottom-up based on a need expressed by prospective parents, the latter being (sometimes) the case in high-risk communities. Literature included in this review raised concerns that couples of the general population may therefore feel opposed to screening or think it is not relevant to them. While interest and intention to partake in EUCS seem to be high when awareness is facilitated, actual uptake in the pilot studies conducted so far seems to be relatively low, as outlined in the systematic review of Steijvoort et al. [65]. Uptake is claimed to be higher when the offer is free of charge or when carrier screening is (also) offered prenatally, with the latter suggesting that awareness is still low preconceptionally, that the perceived personal relevance is higher during pregnancy and/or that relatively more pregnancies are unplanned [65, 66]. Larsen et al. [67], however, reports a partly contrasting finding indicating that uptake is highest when (opportunistically) offered preconceptionally or in early pregnancy. A preconceptional offer combined with a prenatal option would also be most beneficial in terms of equitable access. To facilitate awareness and informed decision-making, proper public education about EUCS is of vital importance.

Dive and Newson [68] argue that public health values, such as equity, should be taken into account when implementing EUCS as well. Articles in the current review also voiced a concern regarding unequitable access to screening, indicating that out-of-pocket costs and inadequate information provision for those who are less (health) literate likely hamper accessibility to EUCS. An important barrier to partaking in screening perceived by members of the general population was also the limited genetic knowledge and awareness about the option of EUCS, and potentially a subsequent lack of perceived benefit among eligible couples [46]. Literature recommends no out-of-pocket costs and adequate information provision and support in decision-making (education and counseling) as ways to improve equitable access [69]. A potential relevant aspect of equitable access in EUCS lacking in the literature identified was access to reproductive options after screening, such as PGT.

## Limitations

This scoping review has several limitations. First, although this review focused on the societal implications of an expanded universal offer, we also included relevant papers referring to the societal implications of high-risk carrier screening offers. We decided upon this approach as these high-risk offers may inform us about which societal implications could be expected in EUCS and we could learn from the empirical evidence from less expanded and non-universal programs. However, our search strategy focused solely on EUCS. Therefore, we probably missed some relevant articles on societal implications of small high-risk offers that would potentially further contribute to our insight into potential societal implications of EUCS, either positive or negative. Second, as this was a scoping review, we aimed to explore all types of data and insights available on societal implications of EUCS. We therefore decided not to limit our inclusion criteria to article types describing primary data. This may have complicated the interpretation of the (little) evidence of articles included in this review. However, we believe that this enabled us to provide an overview of all literature available on this subject. Finally, it was decided to perform a scoping instead of a systematic review, because this better suited our research question. Because of this, we did not perform a quality assessment of the literature reviewed.

## Conclusions

This scoping review aimed to map the potential implications of implementing EUCS on societal level. Importantly, empirical evidence of the prevalence and impact of societal implications of universal offers for society is scarce. Available empirical data is derived from studies on at-risk populations and screening for just one or few disorders. Our findings indicate however several potential positive implications, including increased access for all and less stigmatization of ethnic groups, but also potential negative implications, such as unwanted medicalization of pregnancy, increased stigmatization of and discrimination against people affected with conditions in the test or couples opting out of screening and unequitable access in terms of information provision and costs. More pilot implementation studies assessing EUCS, including their consequences for society, are urgently needed to identify which societal implications occur and to what degree when implementing EUCS.

## References

[1] Fridman H, Yntema HG, Magi R, Andreson R, Metspalu A, Mezzavila M (2021). The landscape of autosomal-recessive pathogenic variants in European populations reveals phenotype-specific effects. Am J Hum Genet.

[2] van der Hout S, Holtkamp KC, Henneman L, de Wert G, Dondorp WJ (2016). Advantages of expanded universal carrier screening: what is at stake?. Eur J Hum Genet.

[3] Molster CM, Lister K, Metternick-Jones S, Baynam G, Clarke AJ, Straub V (2017). Outcomes of an International Workshop on Preconception Expanded Carrier Screening: Some Considerations for Governments. Front Public Health.

[4] Cowan RS (2009). Moving up the slippery slope: mandated genetic screening on Cyprus. Am J Med Genet C Semin Med Genet.

[5] Henneman L, Borry P, Chokoshvili D, Cornel MC, van El CG, Forzano F (2016). Responsible implementation of expanded carrier screening. Eur J Hum Genet.

[6] Raz AE, Vizner Y (2008). Carrier matching and collective socialization in community genetics: Dor Yeshorim and the reinforcement of stigma. Soc Sci Med.

[7] Kalokairinou EM (2008). The experience of beta-thalassaemiaand its prevention in Cyprus. Med Law.

[8] Committee Opinion No. 690 Summary: Carrier Screening in the Age of Genomic Medicine. Obstet Gynecol. 2017;129:595-6. 10.1097/AOG.0000000000001947 28225420

[9] Lazarin GA, Haque IS (2016). Expanded carrier screening: A review of early implementation and literature. Semin Perinatol.

[10] Chokoshvili D, Vears D, Borry P (2018). Expanded carrier screening for monogenic disorders: where are we now?. Prenat Diagn.

[11] Schuurmans J, Birnie E, van den Heuvel LM, Plantinga M, Lucassen A, van der Kolk DM (2019). Feasibility of couple-based expanded carrier screening offered by general practitioners. Eur J Hum Genet.

[12] Ong R, Edwards S, Howting D, Kamien B, Harrop K, Ravenscroft G (2019). Study protocol of a multicentre cohort pilot study implementing an expanded preconception carrier-screening programme in metropolitan and regional Western Australia. BMJ Open.

[13] Dive L, Newson AJ (2021). Ethical issues in reproductive genetic carrier screening. Med J Aust.

[14] Kihlbom U (2016). Ethical issues in preconception genetic carrier screening. Ups J Med Sci.

[15] Richardson E, McEwen A, Newton-John T, Crook A, Jacobs C (2022). Systematic review of outcomes in studies of reproductive genetic carrier screening: Towards development of a core outcome set. Genet Med.

[16] Richardson E, McEwen A, Newton-John T, Crook A, Jacobs C. Correction: Incorporating patient perspectives in the development of a core outcome set for reproductive genetic carrier screening: a sequential systematic review. Eur J Hum Genet. 2022;30:866–67. 10.1038/s41431-022-01090-1 PMC9259674 35347269

[17] Munn Z, Peters MDJ, Stern C, Tufanaru C, McArthur A, Aromataris E (2018). Systematic review or scoping review? Guidance for authors when choosing between a systematic or scoping review approach. BMC Med Res Methodol.

[18] Tricco AC, Lillie E, Zarin W, O’Brien KK, Colquhoun H, Levac D (2018). PRISMA Extension for Scoping Reviews (PRISMA-ScR): Checklist and Explanation. Ann Intern Med.

[19] Janssens A, Gwinn M, Brockman JE, Powell K, Goodman M (2020). Novel citation-based search method for scientific literature: a validation study. BMC Med Res Methodol.

[20] De Wert GM, Dondorp WJ, Knoppers BM (2012). Preconception care and genetic risk: ethical issues. J Community Genet.

[21] Delatycki MB, Alkuraya F, Archibald A, Castellani C, Cornel M, Grody WW (2020). International perspectives on the implementation of reproductive carrier screening. Prenat Diagn.

[22] Ouzzani M, Hammady H, Fedorowicz Z, Elmagarmid A (2016). Rayyan-a web and mobile app for systematic reviews. Syst Rev.

[23] McHugh ML (2012). Interrater reliability: the kappa statistic. Biochem Med (Zagreb).

[24] Popay J, Roberts, H, Sowden, A, Petticrew, M, Arai, L, Rodgers, M, et al. Guidance on the conduct of narrative synthesis in systematic reviews. A product from the ESRC methods programme. 2006. Available from: http://www.lancaster.ac.uk/shm/research/nssr/research/dissemination/publications.php.

[25] Azimi M, Schmaus K, Greger V, Neitzel D, Rochelle R, Dinh T (2016). Carrier screening by next-generation sequencing: health benefits and cost effectiveness. Mol Genet Genom Med.

[26] Beauchamp KA, Johansen Taber KA, Muzzey D (2019). Clinical impact and cost-effectiveness of a 176-condition expanded carrier screen. Genet Med.

[27] Kraft SA, Duenas D, Wilfond BS, Goddard KAB (2019). The evolving landscape of expanded carrier screening: challenges and opportunities. Genet Med.

[28] Langlois S, Benn P, Wilkins-Haug L (2015). Current controversies in prenatal diagnosis 4: pre-conception expanded carrier screening should replace all current prenatal screening for specific single gene disorders. Prenat Diagn.

[29] McGowan ML, Cho D, Sharp RR (2013). The changing landscape of carrier screening: expanding technology and options?. Health Matrix Clevel.

[30] Ready K, Haque IS, Srinivasan BS, Marshall JR (2012). Knowledge and attitudes regarding expanded genetic carrier screening among women’s healthcare providers. Fertil Steril.

[31] Rose NC (2015). Expanded carrier screening: too much of a good thing?. Prenat Diagn.

[32] Wilfond BS, Fost N (1990). The cystic fibrosis gene: medical and social implications for heterozygote detection. JAMA.

[33] Massie J, Ioannou L, Delatycki M (2014). Prenatal and preconception population carrier screening for cystic fibrosis in Australia: where are we up to?. Aust N. Z J Obstet Gynaecol.

[34] Modra LJ, Massie RJ, Delatycki MB (2010). Ethical considerations in choosing a model for population-based cystic fibrosis carrier screening. Med J Aust.

[35] Aspinall PJ (2013). When is the use of race/ethnicity appropriate in risk assessment tools for preconceptual or antenatal genetic screening and how should it be used?. Sociology.

[36] Rowe CA, Wright CF (2020). Expanded universal carrier screening and its implementation within a publicly funded healthcare service. J Community Genet.

[37] Achterbergh R, Lakeman P, Stemerding D, Moors EH, Cornel MC (2007). Implementation of preconceptional carrier screening for cystic fibrosis and haemoglobinopathies: a sociotechnical analysis. Health Policy.

[38] Holtkamp KC, van Maarle MC, Schouten MJ, Dondorp WJ, Lakeman P, Henneman L (2016). Do people from the Jewish community prefer ancestry-based or pan-ethnic expanded carrier screening?. Eur J Hum Genet.

[39] Holtkamp KC, Vos EM, Rigter T, Lakeman P, Henneman L, Cornel MC (2017). Stakeholder perspectives on the implementation of genetic carrier screening in a changing landscape. BMC Health Serv Res.

[40] Holtkamp KCA, Mathijssen IB, Lakeman P, van Maarle MC, Dondorp WJ, Henneman L (2017). Factors for successful implementation of population-based expanded carrier screening: learning from existing initiatives. Eur J Public Health.

[41] Jans SM, van El CG, Houwaart ES, Westerman MJ, Janssens RJ, Lagro-Janssen AL (2012). A case study of haemoglobinopathy screening in the Netherlands: witnessing the past, lessons for the future. Ethn Health.

[42] Lakeman P, Plass AM, Henneman L, Bezemer PD, Cornel MC, ten Kate LP (2008). Three-month follow-up of Western and non-Western participants in a study on preconceptional ancestry-based carrier couple screening for cystic fibrosis and hemoglobinopathies in the Netherlands. Genet Med.

[43] Mathijssen IB, Holtkamp KCA, Ottenheim CPE, van Eeten-Nijman JMC, Lakeman P, Meijers-Heijboer H (2018). Preconception carrier screening for multiple disorders: evaluation of a screening offer in a Dutch founder population. Eur J Hum Genet.

[44] Nijmeijer SCM, Conijn T, Lakeman P, Henneman L, Wijburg FA, Haverman L (2019). Attitudes of the general population towards preconception expanded carrier screening for autosomal recessive disorders including inborn errors of metabolism. Mol Genet Metab.

[45] van der Hout S, Dondorp W, de Wert G (2019). The aims of expanded universal carrier screening: Autonomy, prevention, and responsible parenthood. Bioethics.

[46] Conijn T, van Dijke I, Haverman L, Lakeman P, Wijburg FA, Henneman L (2021). Preconception expanded carrier screening: a focus group study with relatives of mucopolysaccharidosis type III patients and the general population. J Community Genet.

[47] Cornel MC, Rigter T, Jansen ME, Henneman L (2021). Neonatal and carrier screening for rare diseases: how innovation challenges screening criteria worldwide. J Community Genet.

[48] van Dijke I, Lakeman P, Sabiri N, Rusticus H, Ottenheim CPE, Mathijssen IB (2021). Couples’ experiences with expanded carrier screening: evaluation of a university hospital screening offer. Eur J Hum Genet.

[49] Morberg Jamterud S, Snoek A, van Langen IM, Verkerk M, Zeiler K (2021). Qualitative study of GPs’ views and experiences of population-based preconception expanded carrier screening in the Netherlands: bioethical perspectives. BMJ Open.

[50] Ekstrand Ragnar M, Tyden T, Kihlbom U, Larsson M (2016). Swedish parents’ interest in preconception genetic carrier screening. Ups J Med Sci.

[51] Matar A, Hansson MG, Hoglund AT (2019). “A perfect society”- Swedish policymakers’ ethical and social views on preconception expanded carrier screening. J Community Genet.

[52] Matar A, Hansson MG, Hoglund AT (2019). Values and value conflicts in implementation and use of preconception expanded carrier screening - an expert interview study. BMC Med Ethics.

[53] Matar A, Kihlbom U, Hoglund AT (2016). Swedish healthcare providers’ perceptions of preconception expanded carrier screening (ECS)-a qualitative study. J Community Genet.

[54] Janssens S, Chokoshvili D, Vears D, De Paepe A, Borry P (2017). Attitudes of European Geneticists Regarding Expanded Carrier Screening. J Obstet Gynecol Neonatal Nurs.

[55] Frumkin A, Raz AE, Plesser-Duvdevani M, Lieberman S (2011). “The Most Important Test You’ll Ever Take”?: attitudes toward confidential carrier matching and open individual testing among modern-religious Jews in Israel. Soc Sci Med.

[56] Zlotogora J (2009). Population programs for the detection of couples at risk for severe monogenic genetic diseases. Hum Genet.

[57] Zlotogora J, Carmi R, Lev B, Shalev SA (2009). A targeted population carrier screening program for severe and frequent genetic diseases in Israel. Eur J Hum Genet.

[58] Bonneau V, Nizon M, Latypova X, Gaultier A, Hoarau E, Bezieau S (2021). First French study relative to preconception genetic testing: 1500 general population participants’ opinion. Orphanet J Rare Dis.

[59] Bekker H, Modell M, Denniss G, Silver A, Mathew C, Bobrow M (1993). Uptake of cystic fibrosis testing in primary care: supply push or demand pull?. BMJ.

[60] Vanstone M, Cernat A, Majid U, Trivedi F, Freitas CD (2019). Perspectives of Pregnant People and Clinicians on Noninvasive Prenatal Testing: A Systematic Review and Qualitative Meta-synthesis. Ont Health Technol Assess Ser.

[61] Kater-Kuipers A, de Beaufort ID, Galjaard RH, Bunnik EM (2018). Ethics of routine: a critical analysis of the concept of ‘routinisation’ in prenatal screening. J Med Ethics.

[62] Markel H (1992). The stigma of disease: implications of genetic screening. Am J Med.

[63] Muller HJ (1950). Our load of mutations. Am J Hum Genet.

[64] Boardman FK, Clark C, Jungkurth E, Young PJ (2020). Social and cultural influences on genetic screening programme acceptability: A mixed-methods study of the views of adults, carriers, and family members living with thalassemia in the UK. J Genet Couns.

[65] Van Steijvoort E, Chokoshvili D, Cannon JW, Peeters H, Peeraer K, Matthijs G (2020). Interest in expanded carrier screening among individuals and couples in the general population: systematic review of the literature. Hum Reprod Update.

[66] Propst L, Connor G, Hinton M, Poorvu T, Dungan J (2018). Pregnant Women’s Perspectives on Expanded Carrier Screening. J Genet Couns.

[67] Larsen D, Ma J, Strassberg M, Ramakrishnan R, Van den Veyver IB (2019). The uptake of pan-ethnic expanded carrier screening is higher when offered during preconception or early prenatal genetic counseling. Prenat Diagn.

[68] Dive L, Newson AJ. Reproductive carrier screening: responding to the eugenics critique. J Med Ethics. 2021. 10.1136/medethics-2021-107343. 10.1136/medethics-2021-107343 PMC9726954 34244346

[69] Sparks TN (2020). Expanded carrier screening: counseling and considerations. Hum Genet.

